# Thymectomy in Ocular Myasthenia Gravis: Results Before and After Generalization and Prognostic Predictors of Outcomes

**DOI:** 10.3390/jcm14217840

**Published:** 2025-11-04

**Authors:** Dania Nachira, Maria Teresa Congedo, Khrystyna Kuzmych, Amelia Evoli, Raffaele Iorio, Maria Letizia Vita, Leonardo Petracca-Ciavarella, Adriana Nocera, Carolina Sassorossi, Jessica Evangelista, Paraskevas Lyberis, Giovanni Maria Comacchio, Jury Brandolini, Vittorio Aprile, Carmelina Cristina Zifara, Maria Giovanna Mastromarino, Alexandro Patirelis, Elena Asteggiano, Marco Anile, Federico Venuta, Andrea Imperatori, Vincenzo Ambrogi, Piergiorgio Solli, Andrea Dell’Amore, Marco Lucchi, Franca Melfi, Mohsen Ibrahim, Enrico Ruffini, Federico Rea, Stefano Margaritora, Elisa Meacci

**Affiliations:** 1 Department of General Thoracic Surgery, Fondazione Policlinico Universitario “A. Gemelli”, IRCCS, Università Cattolica del Sacro Cuore, 00168 Rome, Italy; dania.nachira@unicatt.it (D.N.); mariateresa.congedo@policlinicogemelli.it (M.T.C.); marialetizia.vita@policlinicogemelli.it (M.L.V.); leonardo.petraccaciavarella@policlinicogemelli.it (L.P.-C.); adriana.nocera91@gmail.com (A.N.); sassorossi.caro@gmail.com (C.S.); stefano.margaritora@unicatt.it (S.M.); elisa.meacci@unicatt.it (E.M.); 2 Institute of Neurology, Fondazione Policlinico Universitario “A. Gemelli”, IRCCS, Università Cattolica del Sacro Cuore, 00168 Rome, Italy; amelia.evoli@unicatt.it (A.E.); raffaele.iorio@policlinicogemelli.it (R.I.); 3 Departmental Unit of Molecular and Genomic Diagnostics, Genomics Core Facility, Gemelli Science and Technology Park (G-STeP), Fondazione Policlinico Universitario “A. Gemelli”, IRCCS, 00168 Rome, Italy; jessica.evangelista@policlinicogemelli.it; 4 Clinical Chemistry, Biochemistry and Molecular Biology Operations (UOC), Fondazione Policlinico Universitario “A. Gemelli”, IRCCS, 00168 Rome, Italy; 5 Department of Thoracic Surgery, University of Torino, 10124 Torino, Italy; paraskevas.lyberis@unito.it (P.L.); enrico.ruffini@unito.it (E.R.); 6 Unit of Thoracic Surgery, Department of Cardiac, Thoracic, Vascular Sciences and Public Health, University of Padova, 35129 Padova, Italy; gcomacchio@gmail.com (G.M.C.); andrea.dellamore@unipd.it (A.D.); federico.rea@unipd.it (F.R.); 7 Division of Thoracic Surgery, IRCCS Azienda Ospedaliera Universitaria Bologna, 40138 Bologna, Italy; jury.brandolini2@unibo.it; 8 Division of Thoracic Surgery, Cardiac, Thoracic and Vascular Department, University Hospital of Pisa, 56124 Pisa, Italy; vittorio.aprile@unipi.it (V.A.); mg.mastromarino@ao-pisa.toscana.it (M.G.M.); marco.lucchi@unipi.it (M.L.); 9 Minimally Invasive and Robotic Thoracic Surgery, Robotic Multispecialty Center of Surgery, University Hospital of Pisa, 56124 Pisa, Italy; c.zirafa@gmail.com; 10 Thoracic Surgery Department, Tor Vergata University Polyclinic, 00133 Rome, Italy; alexandro.patirelis@hotmail.it (A.P.); vincenzo.ambrogi@uniroma2.it (V.A.); 11 Research Center of Minimally Invasive Surgery and Thoracic Surgery, Department of Medicine and Surgery, University of Insubria, 21100 Varese, Italy; elena.asteggiano@asst-settelaghi.it (E.A.); andrea.imperatori@uninsubria.it (A.I.); 12 Department of General and Specialistic Surgeries and Anesthesiology, Policlinico Umberto I, University of Rome Sapienza, 00185 Rome, Italy; marco.anile@uniroma1.it (M.A.); federico.venuta@uniroma1.it (F.V.); 13 Department of Thoracic Surgery, Fondazione IRCCS Istituto Nazionale dei Tumori, 20133 Milan, Italy; piergiorgio.solli@istitutotumori.mi.it; 14 Unit of Thoracic Surgery, Department of Pharmacy and Health and Nutrition Sciences, University of Calabria, 87036 Rende, Italy; franca.melfi@unical.it; 15 Department of Thoracic Surgery, Sant’Andrea Hospital, “Sapienza” University of Rome, 00189 Rome, Italy; mohsen.ibrahim@uniroma1.it

**Keywords:** ocular myasthenia gravis (OMG), thymectomy, complete stable remission (CSR), generalized myasthenia gravis, MGFA-PIS

## Abstract

**Background:** The role of thymectomy in ocular myasthenia gravis (OMG) remains controversial, particularly before secondary generalization. **Methods:** We conducted a multicenter retrospective study on 174 OMG patients who underwent thymectomy (112 OMG, 62 generalized OMG [g-OMG]). The primary endpoint was complete stable remission (CSR; MGFA-PIS criteria). Multivariable analyses identified predictors of CSR and generalization. **Results:** Mean age at surgery was 42.3 ± 13.0 years; 53.4% were male. Thymoma was present in 29.3%. CSR was achieved in 18.9% overall, significantly higher in OMG (23.2%) compared to g-OMG (11.3%, *p* = 0.036), with 5-year CSR probabilities of 43% vs. 22% (*p* = 0.017). In non-thymomatous patients, 5-year CSR remained higher in OMG (41% vs. 17%, *p* = 0.010). Postoperative myasthenic crisis occurred exclusively in g-OMG (8.1%, *p* = 0.004). Multivariable analysis identified preoperative cholinesterase inhibitor monotherapy as an independent predictor of CSR (HR = 31.776, 95% CI: 4.188–241.111, *p* = 0.001; non-thymomatous: HR = 19.746, 95% CI: 2.518–154.849, *p* = 0.005). Minimally invasive techniques (78.6%) were associated with low morbidity (5.2%). **Conclusions:** Thymectomy during the purely ocular stage is associated with higher CSR rates and lower perioperative neurological risk than after generalization, particularly in non-thymomatous disease. Use of cholinesterase inhibitors as sole therapy prior to thymectomy independently predicts CSR. These findings support earlier surgical consideration in selected OMG patients and highlight the safety of minimally invasive approaches.

## 1. Introduction

Myasthenia gravis (MG) is an autoimmune disorder characterized by pathogenic autoantibodies directed against components of the postsynaptic neuromuscular junction, resulting in fluctuating skeletal muscle weakness of variable severity, which is the hallmark clinical manifestation [1]. Based on the distribution of symptoms at disease onset, MG is classified as ocular myasthenia gravis (OMG, MGFA class I) or generalized myasthenia gravis (GMG, MGFA classes II–V) [2]. OMG denotes disease confined to the extraocular muscles (EOMs). Among adult Caucasian MG patients, more than half present initially with ocular symptoms, and approximately 60% subsequently progress to generalized disease (generalized ocular MG, g-OMG), most often within two years of onset. In contrast, up to 20% remain purely ocular throughout the disease course [3,4].

The prognosis of OMG is generally favorable in patients achieving sustained symptom control with low-dose pharmacologic therapy. Immunosuppressive treatment improves ocular symptoms and reduce the risk of secondary generalization [5,6,7,8,9], as suggested by both retrospective and epidemiological. Nevertheless, approximately 35% of patients develop clinically significant adverse effects—including insomnia, gastroesophageal reflux, weight gain, and myalgia—even with low-to-moderate doses of oral corticosteroids [10]. Prognosis is particularly unfavorable in patients with a relapsing course requiring prolonged, high-dose immunosuppression.

Therapeutic options for refractory OMG are limited and primarily supportive, including crutch glasses for severe ptosis, prism lenses for diplopia, surgical correction of ptosis [11,12], topical naphazoline [13], and strabismus surgery [14]. Despite these interventions, recurrence remains common and can substantially complicate the long-term disease course.

Thymectomy is unequivocally indicated in thymomatous MG and, following the results of a landmark randomized controlled trial in 2016 [15,16], is also considered first-line therapy for non-thymomatous GMG. However, its role in OMG remains controversial. The available evidence—derived almost exclusively from retrospective and heterogeneous series—has not yielded definitive conclusions regarding its efficacy in purely ocular disease. Historically, extended transsternal thymectomy was the standard surgical approach, but the diffusion of minimally invasive techniques has made thymectomy more acceptable for selected OMG patients, particularly when an open approach is considered disproportionately aggressive for disease confined to the ocular muscles.

The literature on thymectomy in OMG is inconsistent and, at times, conflicting. Some studies have reported no clear benefit [17,18,19], whereas others suggest that early thymectomy may increase the likelihood of remission and clinical improvement [20,21,22,23]. In current practice, thymectomy is typically considered on a case-by-case basis [24]—either as an initial intervention in early-onset, AChR antibody-positive OMG [25] or in patients with an inadequate response to immunosuppressive therapy [26].

### Objectives

The present multicenter study aimed to evaluate the clinical outcomes of OMG patients undergoing thymectomy before and after secondary generalization. The primary endpoint was complete stable remission (CSR) as defined by the Myasthenia Gravis Foundation of America Post-Intervention Status (MGFA-PIS) criteria. The secondary objective was to identify potential predictors of both generalization and remission following thymectomy in this patient population.

## 2. Materials and Methods

### 2.1. Study Design

This multicenter, retrospective, observational study compared the clinical outcomes of OMG patients undergoing thymectomy before and after secondary generalization. The study was approved by the Ethical Committee of the Università Cattolica del Sacro Cuore (protocol code: 0017919/23; approval date 7 June 2023) and was conducted in accordance with the principles of the Declaration of Helsinki and its subsequent amendments. Data collection and reporting adhered to the Strengthening the Reporting of Observational Studies in Epidemiology (STROBE) guidelines. All patients provided written informed consent for participation and for the anonymous use of their clinical data.

### 2.2. Patient Population

We retrospectively reviewed the clinical records of patients with pure ocular myasthenia gravis (OMG) at disease onset who underwent thymectomy at eight Italian referral centers between January 2000 and January 2023. Data were extracted from each institution’s hospital information system and consolidated into a unified multicenter database.

### 2.3. Inclusion Criteria

Eligible patients were adults with:

1. A confirmed diagnosis of OMG at disease onset, with at least 3 months of isolated ocular symptoms prior to surgery.
2. Thymectomy performed with curative intent.
3. R0 resection in cases of thymoma histology.
4. A minimum postoperative follow-up of 2 years.

### 2.4. Exclusion Criteria

Patients were excluded if they had:

1. Thymic cysts or carcinoma.
2. Prior thymic surgery or thymoma recurrence.
3. Incomplete clinical data or loss to follow-up.

Patients who developed generalized GMG before thymectomy were categorized as the g-OMG group. Those who remained purely ocular at the time of surgery comprised the OMG group. GMG was defined as the presence of symptoms beyond the extraocular muscles or eyelids—such as dysphagia, dysarthria, dyspnea, dysphonia, or limb/neck weakness—confirmed by positive serological or electrophysiological testing.

### 2.5. Diagnosis of OMG

Diagnosis was established by neurologists based on:

- Typical clinical manifestations (fluctuating diplopia, ptosis, or both);
- And at least one of the following:
  1. Positive anti-acetylcholine receptor antibodies (Anti-AChR Ab).
  2. Abnormal repetitive nerve stimulation (RNS).
  3. Positive edrophonium chloride (Tensilon test) or pyridostigmine response.

Patients were stratified according to MGFA classification [27]. Thymectomy was considered on an individual basis in cases of thymic abnormalities suggestive of thymoma, suspicion of hyperplasia with inadequate acetylcholinesterase inhibitor response, early-onset AChR-positive OMG, or unsatisfactory/impossible immunosuppressive management.

### 2.6. Surgical Technique

All patients underwent extended thymectomy in accordance with International Thymic Malignancy Interest Group (ITMIG) recommendations. The procedure included bilateral phrenic nerve and innominate vein identification, with en bloc resection of the thymus and surrounding mediastinal adipose tissue. In cases of suspected thymoma, thorough exploration of perithymic lymph node stations was performed according to the IASLC/ITMIG nodal mapping protocol [28]. Thymomas were resected using a no-touch, en bloc technique to minimize tumor manipulation and preserve oncological integrity [29].

### 2.7. Postoperative Management and Follow-Up

Neurological outcomes were assessed using the Myasthenia Gravis Foundation of America post-intervention status (MGFA-PIS) [30]. Complete stable remission (CSR) was defined as absence of symptoms without medications for ≥1 year.

Patients were followed in neurology outpatient clinics every 3–6 months, with medical therapy adjusted as needed. Patients lost to follow-up were excluded from the analysis.

### 2.8. Study Endpoints

The primary endpoint was CSR rate in the OMG and g-OMG groups.

The secondary endpoints included:

- Identification of predictors of CSR and secondary generalization.
- In the non-thymomatous subgroup: comparison of CSR between OMG and g-OMG and identification of predictors for CSR and generalization.

### 2.9. Statistical Analysis

Continuous variables were reported as mean ± standard deviation if normally distributed or median and interquartile range (IQR) if not normally distributed, while categorical variables as numbers (%). Kolmogorov–Smirnov test was used to evaluate normal distribution of data.

Categorical variables were compared by Chi-squared test or Fischer’s exact test; continuous variables by the independent-sample Student’s *t*-test or the Mann–Whitney U-test, if normally or non-normally distributed.

Kaplan–Meier curves and log-rank test were used to evaluate the estimated cumulative CSR from Thymectomy. Multivariable Cox proportional hazard regression model was used to identify significant predictors of CSR and g-OMG. Only variables with a *p* < 0.2 in the univariable analysis were included in the multivariable analysis.

A *p* < 0.05 was considered statistically significant.

Statistical analysis was performed using the IBM SPSS Statistics for Macintosh, Version 25.00 (Armonk, NY, USA).

## 3. Results

### 3.1. Study Cohort

A total of 174 patients met the inclusion criteria and underwent thymectomy with OMG at disease onset. Among them, 93 (53.4%) were male. The mean age at surgery was 42.28 ± 12.96 years, and the median age at OMG diagnosis was 39 years, so that most patients underwent surgery within a few years of symptom onset.

Before surgery, 62 patients (35.6%) had experiences secondary generalization, forming the g-OMG group, whereas the remaining 112 patients (64.4%) constituted the OMG group.

### 3.2. Baseline Clinical Characteristics

Baseline characteristics are summarized in Table 1. The two groups were generally comparable in terms of demographic and clinical variables with similar sex distribution, mean age at OMG onset, rate of diplopia at onset, age at surgery, and type of surgical approach. Likewise, anti-AChR antibody positivity was observed in 62.5% of OMG and 70.9% of g-OMG patients (*p* = 0.179), showing no statistically significant difference.

In terms of preoperative treatment, significant differences were observed. Azathioprine use was significantly higher among g-OMG patients (16.1% vs. 3.6%, *p* = 0.015). The two groups received comparable proportions of other preoperative medications.

The surgical approach was predominantly robotic-assisted in both groups, around 80%.

With regard to histology, thymoma was more frequent in g-OMG (37.9% vs. 25.0%, *p* = 0.006), whereas thymic hyperplasia predominated in OMG (56.3 vs. 30.6%), yielding a significant group difference (*p* = 0.006). These findings may indicate a distinct pathophysiological backgrounds between early and late generalization.

### 3.3. Perioperative Outcomes

The overall postoperative course was favorable. The mean postoperative hospital stay was 4.42 ± 3.06 days, with a mean chest tube duration of 2.27 ± 2.00 days. A total of nine postoperative complications (5.2%) were recorded, including six Clavien–Dindo grade II and three grade III, all related to chylothorax with one requiring reoperation.

Postoperative intensive care admission during the first 24 h was required in sixty-one patients (35.1%), mainly for precautionary monitoring for potential MG exacerbation. Importantly, there were no perioperative deaths.

### 3.4. Long-Term Outcomes

The mean follow-up duration was 54.49 ± 29.21 months. In the overall cohort, CSR was achieved in 33 patients (18.9%), while a majority showed partial or sustained improvement undergoing medical therapy.

When stratified by disease group (Table 2) CSR was significantly higher in OMG patients than in g-OMG (23.2% vs. 11.3%, *p* = 0.036). Furthermore, no postoperative myasthenic crises occurred in the OMG group, whereas five events (8.1%) were recorded in the g-OMG group (*p* = 0.004), reinforcing the clinical distinction between these populations.

### 3.5. Kaplan–Meier Analysis

**Figure 1:**
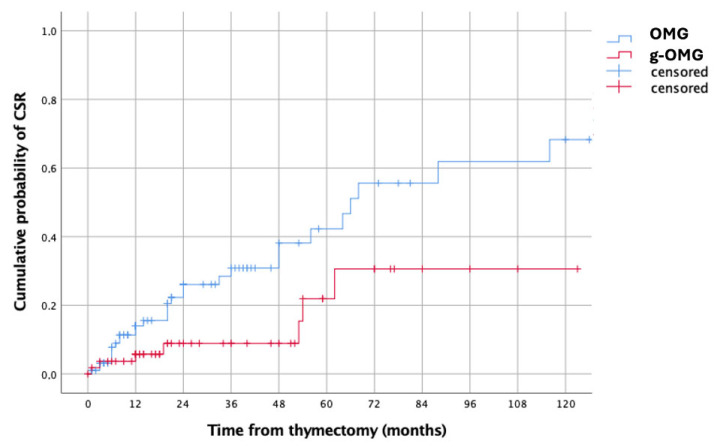
Kaplan–Meier curves showing the estimated cumulative probabilities of CSR after Thymectomy in OMG (blue line) vs. g-OMG (red line) groups.

The estimated 5-year CSR probability was significantly higher in OMG than g-OMG (43% vs. 22%, *p* = 0.017) (Figure 1).

### 3.6. Predictors of CSR

On univariable analysis, preoperative use of cholinesterase inhibitors as monotherapy and azathioprine use showed a significant association with CSR (*p* < 0.001 and *p* = 0.027, respectively). However, in multivariable analysis, only preoperative cholinesterase inhibitors monotherapy resulted as significant independent predictor of CSR in the overall cohort (HR = 31.776, 95% CI: 4.188–241.111, *p* = 0.001) (Table 3).

### 3.7. Predictors of Generalization

Analysis of variables associated with secondary generalization identified thymic hyperplasia as a potential protective factor. Although, it was significant in univariable analysis (*p* = 0.002), it lost significance after adjustment (HR = 0.541, *p* = 0.093) (Table 4).

No other clinical or treatment-related variable independently predicted generalization. A trend toward higher generalization rate was observed in patients with thymoma or in those who discontinued cholinesterase inhibitors before surgery, through without statistical significance.

### 3.8. Subgroup Analysis—Non-Thymomatous MG

**Figure 2:**
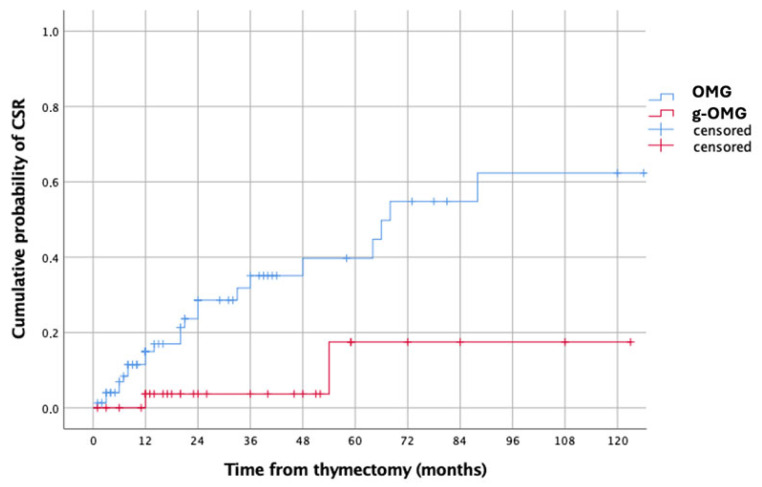
Kaplan–Meier curves showing the estimated cumulative probabilities of CSR after Thymectomy in non-thymomatous OMG (blue line) vs. g-OMG (red line) groups.

Among the 123 patients with non-thymomatous MG, patterns were consistent with the overall cohort. The estimated 5-year CSR was significantly higher in OMG than g-OMG (41% vs. 17%, *p* = 0.010) (Figure 2). Multivariable analysis again identified preoperative use of cholinesterase inhibitors monotherapy as the only significant predictor of CSR (HR = 19.746, 95% CI: 2.518–154.849, *p* = 0.005). No independent predictors of generalization were identified in this subgroup.

## 4. Discussion

Over recent decades, both incidence [31,32,33] and prevalence [34,35] of myasthenia gravis (MG) have consistently increased, a trend mirrored in ocular myasthenia gravis. Among adult Caucasian populations, more than half of MG patients initially present with ocular manifestations, and approximately 60% will progress to generalized MG (g-OMG) within two years of onset; only ~20% remain purely ocular throughout their disease course [3,4]. These epidemiological patterns highlight the clinical importance of clarifying the role of thymectomy in OMG, particularly before secondary generalization occurs.

Minimally invasive thymectomy techniques, including robotic-assisted and video-assisted thoracoscopic surgery (RATS/VATS), have demonstrated comparable, and in some series superior, outcomes to those of extended transsternal thymectomy in selected MG cohorts [36]. This evolution in surgical approach has renewed interest in thymectomy for patients with disease confined to the extraocular muscles. However, high-level evidence supporting thymectomy in OMG remains limited; notably, the only randomized controlled trial of thymectomy in generalized MG [16] excluded patients with purely ocular disease.

### 4.1. Remission Outcomes and Timing of Surgery

In our multicenter series of 174 OMG patients, the overall CSR rate (MGFA-PIS definition) was 18.9%. However, the timing of surgery emerged as a major determinant of outcome. CSR was significantly higher when thymectomy was performed during the purely ocular stage (OMG: 23.2%) compared with after generalization (g-OMG: 11.3%), with estimated 5-year CSR probabilities of 43% and 22%, respectively (*p* = 0.017). This pattern was replicated in the non-thymomatous subgroup (41% vs. 17%, *p* = 0.010).

Postoperative myasthenic crisis occurred exclusively in g-OMG patients (8.1%, *p* = 0.004), underscoring the dual disadvantage of operating after generalization: reduced long-term remission potential and heightened perioperative neurological risk.

These findings parallel those of Li et al. [37], who reported higher 5-year CSR when robotic thymectomy was performed before generalization (49.5% vs. 33.4% overall; 53.5% vs. 28.9% in non-thymomatous patients). Similarly, Liu et al. [38] observed a 5-year CSR of 41.8% in a mixed thymomatous/non-thymomatous OMG cohort, with better outcomes in non-thymomatous cases. In a Chinese series of 110 OMG patients undergoing extended transsternal thymectomy, Liu et al. [20] reported CSR rates of 24.5% at 2 years and 26.4% at 4 years, with higher rates when broader remission definitions were applied. Mineo et al. [21], in a case–control study of non-thymomatous OMG, found that extended thymectomy achieved faster remission than medical therapy alone, particularly when performed within 6 months of symptom onset.

Collectively, these data, along with those of Zhang et al. [39], support a time-sensitive benefit: early thymectomy, particularly in non-thymomatous disease, yields superior remission outcomes. The biological rationale is compelling as generalization reflects broader and more entrenched autoimmune activation, reducing the relative impact of thymic antigen removal.

Our lower overall CSR compared with Zhu et al. [23] is likely explained by two factors:

1. The stringent MGFA-PIS CSR definition used in our study (no symptoms and no therapy for ≥12 months), which yields lower absolute remission rates than broader definitions.
2. Inclusion of patients with thymoma and g-OMG, both associated with more refractory disease, likely diluting the overall CSR signal.

### 4.2. Influence of Thymic Histology

Nearly one-third of our cohort had thymoma, a condition historically associated with more severe MG and reduced remission rates. Several series, including Zhang et al. [39] and Liu et al. [38], have demonstrated that thymic hyperplasia correlates with higher CSR, whereas advanced thymoma predicts worse outcomes. In our data, thymic hyperplasia showed a non-significant protective trend against generalization (HR = 0.541, *p* = 0.093). This suggests that histology may serve as a surrogate marker of disease biology, with hyperplasia more frequently seen in less aggressive forms of MG. The inclusion of thymomatous patients likely contributed to the lower CSR observed in the overall cohort compared with the non-thymomatous subgroup.

### 4.3. Predictors of CSR and Generalization

Multivariable analysis identified preoperative administration of cholinesterase inhibitors as monotherapy as a strong independent predictor of CSR (HR = 31.776, 95% CI: 4.188–241.111, *p* = 0.001 in the overall cohort; HR = 19.746, 95% CI: 2.518–154.849, *p* = 0.005 in non-thymomatous patients). This is unlikely to reflect a direct disease-modifying effect of cholinesterase inhibitors; rather, it probably indicates a subset of patients with a milder phenotype, in whom symptom control was achievable without immunosuppression prior to surgery, inherently increasing the likelihood of meeting strict CSR criteria postoperatively.

### 4.4. Perioperative Safety and Surgical Approach

These figures compare favorably with other contemporary thymectomy series, in which minimally invasive approaches—both video-assisted thoracoscopic surgery (VATS) and robotic-assisted thoracic surgery (RATS)—were associated with reduced blood loss, shorter chest tube duration, and shorter hospital stay, without compromising either oncological integrity or long-term neurological outcomes [40,41]. The increased postoperative crisis risk in g-OMG patients observed in our series is consistent with prior evidence identifying generalized MG at the time of surgery as an independent risk factor for postoperative myasthenic crisis [42,43] further supporting earlier surgical intervention to optimize both neurological and perioperative safety profiles.

Table 5 provides a comprehensive synthesis of published studies evaluating thymectomy in OMG.

### 4.5. Strengths and Limitations

Strengths of this study include its large sample size, the multicenter design encompassing eight high-volume Italian referral centers, the use of standardized MGFA-PIS definitions, and prespecified subgroup analyses by disease stage and histology. Limitations are inherent to the retrospective design, including possible selection and information bias, interinstitutional heterogeneity in diagnostic work-up and perioperative management, and incomplete control for confounding in multivariable analyses. Postoperative medication patterns may also have influenced long-term outcomes. In addition, the study focused exclusively on surgically treated patients, and therefore, no direct comparison with non-surgical management was possible.

Despite these limitations, the present analysis provides valuable multicenter real-world evidence supporting the prognostic relevance of surgical timing and preoperative therapy in OMG patients.

### 4.6. Clinical Implications

For adult OMG patients, our findings support earlier consideration of thymectomy before secondary generalization, particularly in non-thymomatous disease and in suspected thymic hyperplasia, to maximize long-term CSR probability and minimize perioperative neurological risk. After generalization occurs, remission rates are lower and perioperative risk is higher, making the risk–benefit profile less favorable. Minimally invasive approaches appear to offer a favorable safety profile without compromising long-term outcomes.

The clinical heterogeneity of ocular MG likely reflects underlying immunological differences between patients in whom autoimmunity remains confined to ocular muscles and those who later generalize. In early or purely ocular disease, thymic mechanisms such as defective negative selection of autoreactive T cells and ectopic germinal canter formation, are thought to play a predominant role, whereas secondary generalization may result from sustained peripheral T-cell activation and spreading beyond thymic compartment [46]. Interestingly, similar processes have been observed in ICI-induced MG, where loss of immune tolerance and persistent T-cell activation at the neuromuscular junction trigger de novo MG-like syndromes or exacerbating pre-existing disease [47]. These insights support the notion that patient selection for thymectomy should ultimately integrate immunophenotypic markers of thymic immune activity into surgical decision-making.

### 4.7. Future Research Directions

Future prospective, ideally randomized, studies are needed to compare thymectomy versus optimized medical therapy in pure OMG, with stratification by anti-AChR antibody status, thymic histology, and time from symptom onset to surgery. Parallel mechanistic studies incorporating immunological and histopathological biomarkers could clarify whether observed associations reflect causal effects of thymectomy or underlying disease heterogeneity. Treatment resistance in ocular MG may result from heterogeneous disease biology, long disease duration, or suboptimal immunosuppressive response. Further work therefore should investigate potential determinants of treatment resistance, including disease chronicity and interindividual variability in immune regulation. Integrating thymic immunophenotyping and T-cell repertoire profiling could help elucidate how microenvironmental immune alterations influence surgical outcomes. Moreover, comparative research on spontaneous autoimmune MG and immune checkpoint inhibitor-related MG-like syndromes may offer additional insights into shared pathways of immune dysregulation, although these conditions represent distinct clinical entities.

## 5. Conclusions

Thymectomy performed during the purely ocular stage of MG is associated with higher rates of long-term remission and lower perioperative neurological risk compared to surgery after secondary generalization. Minimally invasive approaches may broaden surgical eligibility by reducing procedural morbidity. Preoperative administration of cholinesterase inhibitors as monotherapy emerged as a significant predictor of CSR. These findings provide a strong rationale for earlier surgical consideration in selected OMG patients, particularly in non-thymomatous disease, while underscoring the need for high-quality prospective evidence to refine current practice guidelines.

## Figures and Tables

**Table 1:** Clinical characteristics of the OMG and g-OMG groups.

	OMG (*n* = 112)	g-OMG (*n* = 62)	*p*
**Male sex**	62 (55.4%)	31 (50.0%)	0.459
**Age MG onset (years)**	37.78 ± 13.74	39.07 ± 14.63	0.569
**Age at surgery (years)**	42.99 ± 12.94	41.05 ± 14.10	0.349
**Diplopia onset**	88 (78.6%)	42 (67.7%)	0.310
**Anti-AChR-Ab positive**	70 (62.5%)	44 (70.9%)	0.179
**Preoperative medications**			
Cholinesterase inhibitors	74 (66.1%)	48 (77.4%)	0.225
Corticosteroids	67 (59.8%)	41 (66.1%)	0.168
Azathioprine	4 (3.6%)	10 (16.1%)	**0.015**
**Type of surgery**			0.746
Open	24 (21.4%)	12 (19.4%)	
RATS	88 (78.6%)	50 (80.6%)	
**Thymic histology**			**0.006**
Hyperplasia	63 (56.3%)	19 (30.6%)	
Thymoma	28 (25.0%)	23 (37.9%)	
Involuted thymus	21 (18.8%)	20 (32.3%)	

Data are presented as *n* (%) or mean ± SD unless otherwise specified. OMG = ocular myasthenia gravis; g-OMG = generalized ocular myasthenia gravis; MG = myasthenia gravis; RATS = robotic-assisted thoracoscopic surgery. Only variables with *p* < 0.05 in univariable analysis were included in the multivariable model. Bold values indicate statistical significance (*p* < 0.05).

**Table 2:** Clinical outcomes of OMG and g-OMG groups.

	OMG (*n* = 112)	g-OMG (*n* = 62)	*p*
Post-operative MG crisis	0	5 (8.1%)	**0.004**
**Time to outcomes (months)**	31.99 ± 41.89	32.93 ± 29.13	0.882
**CSR**	26 (23.2%)	7 (11.3%)	**0.036**
**Change in MG status**			**0.036**
Improved	46 (41.1%)	37 (59.7%)	
Unchanged	27 (24.1%)	11 (17.7%)	
worse	13 (11.6%)	7 (11.3%)	
**Postoperative medications**			
Cholinesterase inhibitors	46 (41%)	40 (64.5%)	0.366
Corticosteroids	29 (25.0%)	14 (22.5%)	0.729
Azathioprine	13 (10.7%)	8 (13%)	0.577

CSR = complete stable remission; MG crisis = postoperative myasthenic crisis; “Improved,” “Unchanged,” and “Worse” refer to MGFA post-intervention status compared with preoperative classification. Only variables with *p* < 0.05 in univariable analysis were included in the multivariable model. Bold values indicate statistical significance (*p* < 0.05).

**Table 3:** Predictors of CSR (significant values in bold, *p* < 0.05).

Variables	Univariable Analysis	Multivariable Analysis	
	*p*-Value	HR [95% CI]	*p*-Value
Male sex	0.357		
Age	0.512		
Diplopia	0.597		
Anti-AChR Ab	0.518		
Open surgery	0.404		
Thymoma histology	0.837		
Cholinesterase inhibitors (monotherapy) before surgery	**<0.001**	31.776 [4.188–241.111]	**0.001**
Steroids before surgery	0.237		
Azathioprine before surgery	**0.027**		

Abbreviations: HR = hazard ratio; CI = confidence interval; CSR = complete stable remission. Only variables with *p* < 0.05 in univariable analysis were included in the multivariable model. Bold values indicate statistical significance (*p* < 0.05).

**Table 4:** Predictors of g-OMG (significant values in bold, *p* < 0.05).

Variables	Univariable Analysis	Multivariable Analysis	
	*p*-Value	HR [95% CI]	*p*-Value
Male sex	0.729		
Age	0.468		
Diplopia	0.490		
Anti-AChR Ab	0.309		
Thymic hyperplasia vs. others histologies	**0.002**	0.541 [0.264–1.107]	0.093
Pyridostigmine interruption before surgery	0.646		
Steroids interruption before surgery	0.405		

Abbreviations: HR = hazard ratio; CI = confidence interval; CSR = complete stable remission; g-OMG = generalized ocular myasthenia gravis. Only variables with *p* < 0.05 in univariable analysis were included in the multivariable model. Bold values indicate statistical significance (*p* < 0.05).

**Table 5:** Evidence on Thymectomy in (OMG).

Study	Design	Population & Pathology (OMG)	Surgical Approach	Outcome Definitions	F-Up Months	Key Efficacy Results	Independent Prognostic Factors	P.O Outcomes	Notable Predictors/Insights
Schumm 1985 [44]	RC	18	Transsternal	Improvement/remission	NR	Signal of benefit in OMG	NR	NR	Early series suggesting benefit in OMG
Roberts 2001 [45]	RC	61	Transsternal Transcervical	Improvement/remission	NR	Thymectomy effective and safe in OMG	NR	NR	Early dedicated OMG surgical series
Liu 2011 [20]	RC	110 (5 thymoma)	Transsternal	General CR	Median 33.5	General CR: 41.8% (24 mo), 47.3% (48 mo); strict CR: 24.5% (24 mo), 26.4% (48 mo)	NR	OC 7.8%	Better outcomes in N-T patients
Mineo 2013 [21]	Case–control	47 thymectomy vs. 62 medical	Transsternal	CR; PI	NR	Stable remission: 64% surgical vs. 55% medical;	NR (timing reported as significant).	Major morbidity 4.2%; no mortality	Earlier surgery associated with faster remission
Zhu 2017 [23]	SR-MA (26 studies)	N-T OMG; *n* = 640	Mixed	CSR (heterogeneous definitions)	NA	Pooled CSR ≈ 50% (high heterogeneity) better in children	NA	NA	Highlights definition heterogeneity/regional differences
Hamedani 2020 [19]	RC IPW	30 Transcervical vs. 52 medical	Transcervical	Neuro-ophthalmic remission/improvement	NR	No significant differences	IPW causal weighting	NR	No significant differences
Li 2020 [22]	RC	65 OMG vs. 65 g-Omg N-T subgroup	Robotic	MGFA-aligned CSR	NR	5-yr CSR: 49.5% (OMG) vs. 33.4% (g-OMG); N-T 53.5% vs. 28.9%	Thymectomy in OMG associated with higher CSR	NR	Strong timing effect
Liu 2020 [38]	RC	OMG after thymectomy; *n* = 51 (thymomatous + N-T)	Mixed (extended)	CSR (MGFA-like)	NR	5-yr CSR: 41.8% overall	Age at onset ≤ 40 y → higher CSR (*p* = 0.027)	NR	Younger age favorable
Li 2018 [37]	RC	OMG; risk of generalization	Mixed	Generalization to GMG	Mean 23.6	High conversion overall	Thymoma ↑ generalization (*p* = 0.029). Steroids protective in hyperplasia subgroup (interaction)	NR	Pathology-specific steroid effect
Zhang 2022 [39]	RC	58 OMG post-thymectomy; histology stratified	Mixed	CSR; conversion to GMG	NR	Hyperplasia & stage I thymoma → higher CSR. RNS+ & B2/B3 thymoma → higher conversion	CSR: hyperplasia & stage I thymoma (*p* = 0.026). GMG conversion: RNS+ (*p* = 0.021); B2/B3 thymoma (*p* = 0.048)	NR	RNS-positivity and histotype B2/B3 thymoma: independent predictors of conversion. Thymic hyperplasia and stage I thymoma independently predict CSR.

## Data Availability

The data presented in this study are available on request from the corresponding author.

## References

[1] Gilhus N.E., Tzartos S., Evoli A., Palace J., Burns T.M., Verschuuren J. (2019). Myasthenia gravis. Nat. Rev. Dis. Prim..

[2] Gilhus N.E., Verschuuren J.J. (2015). Myasthenia gravis: Subgroup classification and therapeutic strategies. Lancet Neurol..

[3] Luchanok U., Kaminski H.J. (2008). Ocular myasthenia: Diagnostic and treatment recommendations and the evidence base. Curr. Opin. Neurol..

[4] Wong S.H., Huda S., Vincent A., Plant G.T. (2014). Ocular myasthenia gravis: Controversies and updates. Curr. Neurol. Neurosci. Rep..

[5] Benatar M., Kaminski H. (2012). Medical and surgical treatment for ocular myasthenia. Cochrane Database Syst. Rev..

[6] Bever C.T., Aquino A.V., Penn A.S., Lovelace R.E., Rowland L.P. (1983). Prognosis of ocular myasthenia. Ann. Neurol..

[7] Kupersmith M.J., Moster M., Bhuiyan S., Warren F., Weinberg H. (1996). Beneficial effects of corticosteroids on ocular myasthenia gravis. Arch. Neurol..

[8] Sommer N., Sigg B., Melms A., Weller M., Schepelmann K., Herzau V., Dichgans J. (1997). Ocular myasthenia gravis: Response to long-term immunosuppressive treatment. J. Neurol. Neurosurg. Psychiatry.

[9] Oosterhuis H.J. (1989). The natural course of myasthenia gravis: A long-term follow-up study. J. Neurol. Neurosurg. Psychiatry.

[10] Lee Y.G., Kim U.S. (2018). Efficacy and safety of low-to-moderate dose oral corticosteroid treatment in ocular myasthenia gravis. J. Pediatr. Ophthalmol. Strabismus.

[11] Farrugia M.E., Goodfellow J.A. (2020). A practical approach to managing patients with myasthenia gravis—Opinions and a review of the literature. Front. Neurol..

[12] Shimizu Y., Suzuki S., Nagasao T., Ogata H., Yazawa M., Suzuki N., Kishi K. (2014). Surgical treatment for medically refractory myasthenic blepharoptosis. Clin. Ophthalmol..

[13] Nagane Y., Utsugisawa K., Suzuki S., Masuda M., Shimizu Y., Utsumi H., Uchiyama S., Suzuki N. (2011). Topical naphazoline in the treatment of myasthenic blepharoptosis. Muscle Nerve.

[14] Smith S.V., Lee A.G. (2017). Update on ocular myasthenia gravis. Neurol. Clin..

[15] Sonett J.R., Magee M.J., Gorenstein L. (2017). Thymectomy and myasthenia gravis: A history of surgical passion and scientific excellence. J. Thorac. Cardiovasc. Surg..

[16] Wolfe G.I., Kaminski H.J., Aban I.B., Minisman G., Kuo H.-C., Marx A., Ströbel P., Mazia C., Oger J., Cea J.G. (2016). Randomized trial of thymectomy in myasthenia gravis. N. Engl. J. Med..

[17] Evoli A., Batocchi A.P., Provenzano C., Ricci E., Tonali P. (1988). Thymectomy in the treatment of myasthenia gravis: Report of 247 patients. J. Neurol..

[18] Hatton P.D., Diehl J.T., Daly B.D.T., Rheinlander H.F., Johnson H., Schrader J.B., Bloom M., Cleveland R.J. (1989). Transsternal radical thymectomy for myasthenia gravis: A 15-year review. Ann. Thorac. Surg..

[19] Hamedani A.G., Pistilli M., Singhal S., Shindler K.S., Avery R.A., Tamhankar M.A., Liu G.T. (2020). Outcomes after transcervical thymectomy for ocular myasthenia gravis: A retrospective cohort study with inverse probability weighting. J. Neuroophthalmol..

[20] Liu Z., Feng H., Yeung S.-C.J., Zheng Z., Liu W., Ma J., Zhong F.-T., Luo H., Cheng C. (2011). Extended transsternal thymectomy for the treatment of ocular myasthenia gravis. Ann. Thorac. Surg..

[21] Mineo T.C., Ambrogi V. (2013). Outcomes after thymectomy in class I myasthenia gravis. J. Thorac. Cardiovasc. Surg..

[22] Li F., Li Z., Chen Y., Bauer G., Uluk D., Elsner A., Swierzy M., Ismail M., Meisel A., Rückert J.-C. (2020). Thymectomy in ocular myasthenia gravis before generalization results in a higher remission rate. Eur. J. Cardiothorac. Surg..

[23] Zhu K., Li J., Huang X., Xu W., Liu W., Chen J., Chen P., Feng H. (2017). Thymectomy is a beneficial therapy for patients with nonthymomatous ocular myasthenia gravis: A systematic review and meta-analysis. Neurol. Sci..

[24] Melzer N., Ruck T., Fuhr P., Gold R., Hohlfeld R., Marx A., Melms A., Tackenberg B., Schalke B., Schneider-Gold C. (2016). Clinical features, pathogenesis, and treatment of myasthenia gravis: A supplement to the guidelines of the German Neurological Society. J. Neurol..

[25] Sussman J., Farrugia M.E., Maddison P., Hill M., Leite M.I., Hilton-Jones D. (2015). Myasthenia gravis: Association of British Neurologists’ management guidelines. Pract. Neurol..

[26] Kerty E., Elsais A., Argov Z., Evoli A., Gilhus N.E. (2014). EFNS/ENS Guidelines for the treatment of ocular myasthenia. Eur. J. Neurol..

[27] Jaretzki A., Barohn R.J., Ernstoff R.M., Kaminski H., Keesey J., Penn A., Sanders D. (2000). Myasthenia gravis: Recommendations for clinical research standards. Task Force of the Medical Scientific Advisory Board of the Myasthenia Gravis Foundation of America. Neurology.

[28] Ruffini E., Filosso P.L., Guerrera F., Lausi P., Lyberis P., Oliaro A. (2018). Optimal surgical approach to thymic malignancies: New trends challenging old dogmas. Lung Cancer.

[29] De Iaco G., Brascia D., Geronimo A., Sampietro D., Fiorella A., Schiavone M., Panza T., Signore F., Marulli G. (2020). Standardized definitions and concepts of radicality during minimally invasive thymoma resection. Mini-Invasive Surg..

[30] Girard N., Ruffini E., Marx A., Faivre-Finn C., Peters S. (2015). ESMO Guidelines Committee. Thymic epithelial tumours: ESMO Clinical Practice Guidelines for diagnosis, treatment and follow-up. Ann. Oncol..

[31] Maddison P., Ambrose P.A., Sadalage G., Vincent A. (2019). A prospective study of the incidence of myasthenia gravis in the East Midlands of England. Neuroepidemiology.

[32] Lotan I., Benninger F., Hellmann M.A., Sicsic C., Brenner T., Kahana E., Steiner I. (2020). Incidence of AChR Ab-positive myasthenia gravis in Israel: A population-based study. Acta Neurol. Scand..

[33] Gattellari M., Goumas C., Worthington J.M. (2012). A national epidemiological study of myasthenia gravis in Australia. Eur. J. Neurol..

[34] Breiner A., Widdifield J., Katzberg H.D., Barnett C., Bril V., Tu K. (2016). Epidemiology of myasthenia gravis in Ontario, Canada. Neuromuscul. Disord..

[35] Boldingh M.I., Maniaol A.H., Brunborg C., Dekker L., Heldal A.T., Lipka A.F., Popperud T.H., Niks E.H., Verschuuren J., Tallaksen C.M. (2015). Geographical distribution of myasthenia gravis in northern Europe—Results from a population-based study from two countries. Neuroepidemiology.

[36] Narayanaswami P., Sanders D., Wolfe G., Benatar M., Cea G., Evoli A., Gilhus N.E., Illa I., Kuntz N.L., Massey J. (2021). International Consensus Guidance for Management of Myasthenia Gravis: 2020 Update. Neurology.

[37] Li F., Hotter B., Swierzy M., Ismail M., Meisel A., Rückert J.C. (2018). Generalization after ocular onset in myasthenia gravis: A case series in Germany. J. Neurol..

[38] Liu X., Zhou W., Hu J., Hu M., Gao W., Zhang S., Zeng W. (2020). Prognostic predictors of remission in ocular myasthenia after thymectomy. J. Thorac. Dis..

[39] Zhang J., Zhang Z., Zhang H., Cui Y., Chen Y., Lv P., Zhang P. (2022). Thymectomy in ocular myasthenia gravis—Prognosis and risk factors analysis. Orphanet J. Rare Dis..

[40] Sudarshan M., Raja S. (2021). Re-operative surgery after paraesophageal hernia repair: Narrative review. Video Assist. Thorac. Surg..

[41] Friedant A.J., Handorf E.A., Su S., Scott W.J. (2016). Minimally Invasive versus Open Thymectomy for Thymic Malignancies: Systematic Review and Meta-Analysis. J Thorac Oncol..

[42] Wei B., Lu G., Zhang Y. (2023). Predictive factors for postoperative myasthenic crisis in patients with myasthenia gravis. Interdiscip. Cardiovasc. Thorac. Surg..

[43] Ruan Z., Su Y., Tang Y., Lin J., Lu Q., Zhou Y., Guo R., Liu Y., Li H., Sun C. (2023). Nomogram for predicting the risk of postoperative myasthenic crisis in patients with thymectomy. Ann. Clin. Transl. Neurol..

[44] Schumm F., Wiethölter H., Fateh-Moghadam A., Dichgans J. (1985). Thymectomy in myasthenia with pure ocular symptoms. J. Neurol. Neurosurg. Psychiatry.

[45] Roberts P.F., Venuta F., Rendina E., De Giacomo T., Coloni G.F., Follette D.M., Richman D.P., Benfield J.R. (2001). Thymectomy in the treatment of ocular myasthenia gravis. J. Thorac. Cardiovasc. Surg..

[46] He T., Chen K., Zhou Q., Cai H., Yang H. (2024). Immune repertoire profiling in myasthenia gravis. Immunol. Cell Biol..

[47] Marco C., Simó M., Alemany M., Casasnovas C., Domínguez R., Vilariño N., Calvo M., Martín-Liberal J., Brenes J., Sabater-Riera J. (2022). Myasthenia Gravis Induced by Immune Checkpoint Inhibitors: An Emerging Neurotoxicity in Neuro-Oncology Practice: Case Series. J. Clin. Med..

